# Whether the start time of elective lung surgery impacts perioperative outcomes and cost?

**DOI:** 10.3389/fsurg.2022.922198

**Published:** 2022-08-24

**Authors:** Gu-Ha A-Lai, Jian-Rong Hu, Zhi-Jie Xu, Peng Yao, Xia Zhong, Yu-Cheng Wang, Yi-Dan Lin

**Affiliations:** ^1^ Department of Thoracic Surgery, West China Hospital, Sichuan University, Chengdu, China; ^2^ Operating Room of Anesthesia Surgery Center, West China Hospital/West China School of Nursing, Sichuan University, Chengdu, China

**Keywords:** start time, perioperative outcome, cost, after-hours, elective lung surgery

## Abstract

**Background:**

Fatigue and the long work hours of surgeons have attracted increasing concern in recent years. We aimed to explore whether starting time was associated with perioperative outcomes and cost for elective lung surgery.

**Methods:**

A retrospective study was conducted on elective lung surgery patients at a high surgery-volume center between September 2019 and November 2019. Patients were divided into the “early start group” if the surgery start time was before 4 post meridiem (pm), while the “late start group” was defined as surgery started after 4 pm. Perioperative outcomes and total hospital costs were compared between the two groups. In addition, multivariable logistic regression analysis was performed to identify whether start time was a risk factor for postoperative hospital duration, total hospital cost and length of operation time.

**Results:**

A total of 398 patients were finally enrolled for analysis in this study. Of all the cases, 295 patients were divided into the early start group, while 103 patients belonged to the late start group. Baseline characteristics were all comparable between the two groups. Concerning Regarding outcomes, there were no differences in postoperative hospital duration, operation time, complication incidence or and other outcomes, while the total hospital cost tended to be different but still not significantly different without statistical significance (*P *= 0.07). In multivariable logistic regression analysis, surgery starting late was still not found to be a risk factor for long postoperative hospital duration, high hospital cost and long surgery time.

**Conclusion:**

In elective lung surgery, perioperative outcomes and costs were similar between the early- and late-start groups, and it was not necessary to worry about the surgery order for these patients.

## Introduction

In general, fatigue and sleep deprivation are thought to result in low-efficiency work and even mistakes. There is a consensus that circadian rhythm disturbances, overloaded work and fatigue could significantly increase accidents in the transportation industry (1). How about the impact in medical domain field? Death of Libby Zion, a widely publicized case, created giant social influence and then motivated the revolution of resident education in America because of the resident physician was found to be in a long-term overload work state (2). The impacts of fatigue and sleep deprivation of physicians attract increasing concern, and a well-documented noun called the “after-hour” effect is created that combines patient handoffs, resource limitations and differences in the expertise of hospital staff after long-duration work or in weekend time (3). A study including surgical residents found that fatigue and deprivation of sleep decreased psychomotor and cognitive skills (4). However, clinical studies investigating the impacts of after-hour care have not reached a consensus; some conclude that late-start surgery in the day could increase complication morbidity, mortality, cost and length of hospital stay (5–9), while others have found no associations between outcomes and surgery start time (10–13).

It is known that the number of lung surgeries has have rapidly increased in recent years, with an increasing number of small nodules identified in China. However, there is only one study finding that video-assisted lobectomy conducted in the late week could increase the length of hospital stay (9). Impacts of late start in the day still remain unclear concerning elective lung surgery. Most Chinese people have the habit of sleeping at noon, and many official departments obey the rule of 8-hour work, while a surgeon could discard both rules during a surgery day and which resulted in deprivation of sleep and fatigue after 4:00 pm. In addition, many Chinese patients always require surgeons to arrange to receive surgery first. Their routine thoughts are that surgeons are more energetic in the morning, but thoracic surgeons always think they can provide the same high-quality medical service for all patients. Under this circumstance, it was extremely proper in urgent need to conduct this study to testify our hypothesis that late-start lung surgery for patients was not associated with negative outcomes and provide an answer for anxious patients.

## Methods

### Patients

All patients received elective lung surgery were retrospectively enrolled between September 2019 and November 2019 at the Department of Thoracic Surgery, West China Hospital. Inclusion criteria: (1) lung surgery; (2) elective surgery; (3) age ≥18 years old; (4) normal preoperative renal and liver function. Exclusion criteria: (1) clinical data were sufficient; (2) lung surgery combined with other organ surgery; (3) combined surgery (wedge resection, segmentectomy and lobectomy); (4) urgent or emergent surgery of patients intended to receive elective surgery during hospitalization. All patient information cnilical data was extracted from the electronic medical records system. Our institutional review board approved this study, and patient consent was waived owing to the retrospective nature of this study.

### Study design and definitions

The point of skin incision making was viewed as the start time of one surgery. According to this definition of start time, surgery started between 8 ante meridiem (am) and 4 pm was defined as “early start”, while “late start”, namely, started after 4 pm for elective surgery. The start time of all the surgeries was between 8 am and 9 am in our hospital according to hospital rules and necessary preparation. One rule concerning elective surgery in our hospital is that surgery of the one case before the last case must be completed before 7 pm unless the last case could not be received by the operating room in one day. All the surgeons in this study had abundant lung surgery experience and had performed lung surgery at least 400 cases and at least 5 years before this study. In this study, one surgeon conducted all the surgeries without changing surgeon all the day. Primary outcomes included postoperative hospital duration and total hospital cost, and these data could be collected in the electronic medical records system. Secondary outcomes included operation time, duration of chest tube placement, intraoperative bleeding volume, intraoperative conversion, duration of intensive care unit (ICU) stay, complications (lung infection, pulmonary embolism, stroke, postoperative bleeding, incision infection, chylothorax, bronchial fistula, hoarseness, prolonged air leak, other site infection) and operative mortality (death occurred postoperatively in the hospital or within 30 days after surgery). Prolonged air leak was defined as >5 days. All the data were also collected from the hospital electronic medical records system by two doctors (Hu JR and Yao P), while definitions for postoperative events and complications were according to the Society of Thoracic Surgeons and the European Society of Thoracic Surgeons.

### Statistical analysis

Statistical analysis was performed using SPSS 26.0 software (SPSS Corp., Chicago, IL, United States). Concerning continuous data, the mean ± standard deviation was adopted for normally distributed continuous variables, while nonnormally distributed continuous variables were demonstrated with the median [interquartile range]. Categorical variables are shown as number. Student's t test or the Mann–Whitney *U* test was used to compare continuous variables, while the Chi (2) test or Fisher's exact test was used for categorical variables according to specific data. Because all the baseline characteristics were comparable, we omitted the application of the statistical method of propensity-scored matching. And patients were divided into two groups according to the median value for continous variables which included cost and others. Then, a multivariable logistic regression model was accepted for risk factor analysis, and we included variables with a *P* value ≤0.15 in the baseline characteristics analysis, surgery start time and other probable clinical factors in multivariable analysis. Odds ratios (ORs) are presented with 95% confidence intervals (CIs) for logistic regressions. Multivariable analysis for postoperative complications was omitted owing to the small sample size of only 25 patients who suffered complications. A *P* value of <0.05 was considered statistically significant, which was based on a two-sided test.

## Results

### Patient baseline and outcome comparison

A total of 398 patients were finally enrolled for analysis according to the inclusion and exclusion criteria between September 2019 and November 2019 at our department. Among these, And 295 patients received lung surgery before 4 pm, while other cases in the group received lung surgery after 4 pm. As shown in Table 1, all the baseline characteristics, including continuous variables and categorical variables, were comparable between the early-start group and the late-start group. Only diabetes incidence seemed to be higher in the early start group, but without statistical significance, the incidence of total comorbidity was also comparable between the two groups. All outcomes are were shown in Table 2. Regarding the primary outcome, there was no difference in postoperative hospital duration (*P* = 0.363), and the total hospital cost tended to be higher in the early start group, but the difference was not statistically significant (*P* = 0.07). In addition, concerning regarding the second outcome, no differences were found in operation time, intraoperative bleeding volume, length of ICU time, length of chest tube time, complication incidence or conversion. Among all the patients, no deaths occurred within 30 days after surgery, and only one patient received a transfusion in the early start group.

**Table 1:** Baseline characteristics comparison between the early-stage and late-stage groups.

Variables	Early start group (*n* = 295)	Late start group (*n* = 103)	*P*
Age	56 (49, 65)	56 (48, 65)	0.771
BMI	22.66 (20.96, 24.52)	22.89 (21.09, 25.14)	0.442
Tumor size	1.4 (1, 2.1)	1.2 (0.9, 2.0)	0.162
Number of lymph nodes	5 (4, 6)	5 (3, 6)	0.364
Gender			0.462
Male	121	38	
Female	174	65	
Smoking			0.781
Yes	64	21	
No	231	82	
Alcohol			
Yes	48	20	
No	247	83	
Comorbidities			0.788
Yes	93	31	
No	202	72	
Hypertension			0.959
Yes	68	24	
No	227	79	
Diabetes			0.072
Yes	28	4	
No	267	99	
Coronary artery disease			0.736
Yes	20	8	
No	275	95	
COPD			0.763
Yes	10	4	
No	285	99	
Kidney dysfunction			0.451
Yes	1		
No	294		
Liver dysfunction			1.000
Yes	2	0	
No	293	103	
Surgical range			0.335
Lobectomy	168		
Segmentectomy	83		
Wedge resection	44		
Surgical method			0.651
Minimally invasive surgery	291	101	
Open surgery	4	2	
Surgical approach			0.791
VATS	234	85	
UVATS	52	15	
RATS	5	1	
Open	4	2	
Histology			0.599
Benign	28	8	
Malignant	267	95	
ASA grade			0.316
1	2	2	
2	239	86	
3	54	15	

BMI, body weight index; ASA, American society of anesthesiology; VATS, video-assisted thoracoscopic surgery; UVATS, uniportal video-assisted thoracoscopic surgery; RATS, robot-assisted thoracoscopic surgery.

**Table 2:** Outcome comparison between the early-stage and late-start groups.

Variables	Early start group (*n* = 295)	Late start group (*n* = 103)	*P*
Hospital duration	4 (3, 5)	4 (3, 5)	0.363
Cost (CNY)	52,967.43 (46,966.06, 61,862.49)	51,217.93 (45,122.32, 56,414.69)	0.07
Operation time	90 (70, 120)	86 (60, 119)	0.408
Chest tube time	2 (2, 4)	2 (2, 3)	0.992
Intraoperative bleeding	20 (20, 30)	20 (20, 50)	0.356
ICU time	0 (0, 0)	0 (0, 0)	0.682
Complications			0.792
Yes	19	6	
No	276	99	
Lung infection			0.701
Yes	6	3	
No	289	100	
Air leakage			0.528
Yes	11	2	
No	284	101	
Chylothorax		1.000	
Yes	2	1	
No	293	102	
Transfusion			–
Yes	1	0	
No	294	103	
Conversion		0.608	
Yes	3	2	
No	292	101	
Mortality			–
Yes	0	0	
No	295	103	

CNY, chinese yuan; ICU, intensive care unit.

### Multivariable postoperative hospital duration analysis

All the patients were divided into two groups according to the median postoperative hospital duration, and a univariable comparison is shown in **Supplementary Material 1**. Then, we conducted multivariable logistic analysis and found that late start was not associated with increased postoperative hospital duration, with an OR of 0.715 (95% Cl, 0.400–1.275, *P* = 0.255). In addition, there were no associations between postoperative hospital duration and comorbidity, operation time, surgical range or surgical method. However, patients who expended a higher total hospital cost had a significantly longer postoperative hospital duration, with an OR of 4.498 (95% Cl, 2.689–7.498, *P* < 0.001). All are shown in Table 3.

**Table 3:** Multivariable logistic regression analysis of postoperative hospital duration.

Variables	OR	95%Cl	*P*
Time of surgery			
Early start	1	–	–
Late start	0.715	0.400–1.275	0.255
Comorbidity			
No	1	–	–
Yes	1.351	0.805–2.269	0.255
Surgical range			
Wedge	1	–	–
Lobe	1.087	0.519–2.280	0.825
Segment	0.579	0.254–1.320	0.194
Surgical method			
Open	1	–	–
MIS	0.410	0.029–5.836	0.511
Operation time(min)			
≤89	1	–	–
>89	1.581	0.951–2.628	0.077
Cost (CNY)			
≤53,060	1	–	–
>53,060	4.498	2.689–7.498	<0.001

OR, odds ratio; 95% CI, 95% confidence interval; MIS, minimally invasive surgery.

### Multivariable cost analysis

All the patients were divided into two groups according to the median total hospital cost. A univariable comparison is was shown in **Supplementary Material 2**. High hospital cost was associated with late-start surgery, longer postoperative hospital duration, longer drainage time, suffering complications and comorbidities. However, as the results of multivariable analysis demonstrated in Table 4, late start was not associated with increased total hospital cost, with an OR of 0.741 (95% Cl, 0.444–1.239, *P* = 0.253). Comorbidity status, complication status and sex were also not associated with high total hospital cost. Only a longer postoperative hospital duration and chest tube duration significantly increased the total hospital cost, with ORs of 2.779 (95% Cl, 1.580–4.955, *P* < 0.001) and 2.347 (95% Cl, 1.410–3.977, *P* = 0.001), respectively.

**Table 4:** Multivariable logistic regression analysis of total hospital cost.

Variables	OR	95%Cl	*P*
Time of surgery			
Early start	1	–	–
Late start	0.741	0.444–1.239	0.253
Comorbidity			
No	1	–	–
Yes	1.149	0.691–1.912	0.592
Smoking			
No	1	–	–
Yes	1.075	0.513–2.253	0.847
Alcohol			
No	1	–	–
Yes	1.062	0.510–2.213	0.872
Gender			
Male	1	–	–
Female	0.605	0.336–1.091	0.095
Postoperative duration (day)			
≤4	1	–	–
>4	2.779	1.580–4.955	<0.001
Chest tube duration			
≤2	1	–	–
>2	2.374	1.410–3.997	0.001

### Multivariable operation time analysis

Finally, all the patients were divided into two groups according to the median operation time. A univariable comparison is was shown in **Supplementary Material 3**, and only more lymph node dissection was were significantly associated with a longer surgical time. In Table 5, multivariable logistic analysis demonstrated late start surgery did not impact operation time 0.927 (95% Cl, 0.575–1.492, *P* = 0.754), so did variables of age, number of lymph nodes dissection and histology. However, lobectomy and segmentectomy significantly increased the operation time, with ORs of 5.004 (95% Cl, 2.428–10.310, *P* < 0.001) and 3.467 (95% Cl, 1.661–7.233, *P* < 0.001), respectively, compared with wedge resection.

**Table 5:** Multivariable logistic regression analysis of operation time.

Variables	OR	95%Cl	*P*
Time of surgery			
Early start	1	–	–
Late start	0.927	0.575–1.492	0.754
Surgical range			
Wedge resection	1	–	–
Lobectomy	5.004	2.428–10.310	<0.001
Segmentectomy	3.467	1.661–7.233	<0.001
Age			
≤56	1	–	–
>56	0.982	0.646–1.495	0.934
Number of lymph nodes			
≤5	1	–	–
>5	0.978	0.626–1.527	0.922
Histology			
Malignant	1	–	–
Benign	0.744	0.342–1.620	0.457

VATS, video-assisted thoracoscopic surgery; UVATS, uniportal video-assisted thoracoscopic surgery; RATS, robot-assisted thoracoscopic surgery.

## Discussion

To the best of our knowledge, few studies have explored the outcomes of elective lung surgery patients and start time, while many studies have explored the relationship between other organ surgeries and outcomes. In this retrospective, high surgery-volume center study of the impact of start time and surgery outcomes, we found no difference in the early start group and late start group concerning regarding short outcomes and total hospital cost. In addition, we also revealed that a late start was not a risk factor for a longer postoperative duration, higher total hospital cost or longer operation time. This result was completely consistent with our primary hypothesis. The complication incidence of lung surgery patients was decreased to an extremely low level in high surgery-volume centers by all kinds of perioperative elaborate management, and only 25 patients suffered postoperative complications in this study. Hence, we omitted the need to conduct multivariable analysis for postoperative complications owing to the extremely low sample size. For the cutoff time, we selected 4 pm as in some previous studies (7, 10, 14). Another reason for the selection of this time was that related labor law defines a routine work duration of 8 h, and surgeons begin work 8 am in our hospital.

For the most common question of patients before surgery, “May I be the first one to receive surgery in surgery order of tomorrow?” Now, we could definitely answer “it is not necessary” loudly from the results of this study. All the outcomes and total hospital cost were comparable between the two groups. There are several possible reasons, including that surgeons could work continually with strong will and have a rest between surgery intervals, and all the surgery team could keep concentrating on patient status, ensuring surgery safely. Among the baseline characteristic comparisons between the two groups, all the variables were comparable, and the propensity-scored match procedure was omitted. Among the outcomes, no significant differences were found in any of the variables. In addition, it is was interesting that the total hospital cost in the early start group was prone to be higher than that in the late start group, while the difference was not statistically significant. This result furtherly proved that late start for elective lung surgery did not have any negative impact even toward cost less than compared starting early, which strongly supported our primary hypothesis. Similarly, surgery start time did not impact cost, postoperative hospital duration or operation time in multivariable analysis, although start late was associated with high cost and prone to result in longer postoperative hospital duration in single variable analysis. In summary, no association was found between elective lung surgery stat time and any outcome.

In elective thoracic surgery, Bao and his colleagues also found that a later start time had no impact on either short- or long-term outcomes for patients undergoing minimally invasive McKeown esophagectomy *via* a propensity-scored match study (11). In addition, studies concerning regarding elective cardiac surgery also find no association between start time and perioperative outcomes, operative mortality, length of stay and total hospital cost (10, 12, 13, 15). The studies above all agree with the same conclusion as this study, possibly due to a reasonable shift of assisted staff, a sufficient supply of surgery-related materials and the surgeons' strong will and concentration. However, some different voices do exist. A study that enrolled 208 patients found that lobectomy conducted late in the week significantly increased the length of hospital stay compared with early in the week (9). Yount et al. also found that late-start cardiac surgery increases absolute and risk-adjusted mortality (5). Another study enrolled more than 100000 patients and demonstrated that the incidence of anesthetic adverse events is higher in the late start group (*P* < 0.0001), although bias existed in the study (7). Similar to these studies, a study from the New England Journal of Medicine found find that patients with serious diseases had higher mortality when they were admitted on weekend days than on weekdays (16). In addition, medical education studies of fatigue and sleep deprivation have also found that the surgical skills of residents could be affected by these unfavorable situations (4, 17). These studies believe these unfavorable outcomes resulted from all aspects, including fatigue of both surgeons, anesthetists and nurses and lack of sources. However, in large medical centers such as our center, sources of materials supply will always be sufficient, and nurses and anesthetists also have proper shifts at fixed times, so outcomes in different levels of hospitals could also be different.

There were several limitations in this study. First, as a retrospective study, inherent shortcomings could decrease the reliability degree of evidence. Second, a sample of approximately 400 patients was relatively small, and more multicenter, large sample and prospective studies could be beneficial in the future. Third, the incidence of postoperative complications was too low to perform further analysis of the relationship with the start time of lung surgery. Fourth, patients enrolled in this study contained different histology and different surgery approaches which could result in bias, but the variables were comparable in different groups, which made the results still reliable. Finally, this study only enrolled elective lung surgery, and the association start time and urgent or emergent lung surgery need further investigation in the future.

## Conclusion

In this study, we demonstrated that there were no differences in postoperative hospital duration, hospital cost, operation time, complication incidence or and postoperative mortality between surgery started before and after 4 pm. In addition, surgery started late was identified as having no risk factors for outcomes by multivariable analysis. Hence, this study suggested that patients could wait for surgery silently without any worry. and provided evidence for surgeons to properly increase the surgery amount in high-volume surgery centers.

## Data Availability

The original contributions presented in the study are included in the article/**Supplementary Material**, further inquiries can be directed to the corresponding author/s.
