# Supplementary material for: Whether the start time of elective lung surgery impacts perioperative outcomes and cost?
Source: Front Surg. 2022 Aug 24;9:922198. doi: 10.3389/fsurg.2022.922198 (PMC9448901; doi:10.3389/fsurg.2022.922198)
Supplement: Supplementary file 1 [file Table_3_v1.docx]

Supplement material 1. Baseline characteristics comparison between short and long postoperative duration group.

| Variables short stay group (n=262) long stay group (n=136) P |
| --- |
| Age 56(47, 64) 59(51, 68) 0.479  BMI 22.84(21.07, 24.54) 22.40(20.81, 24.57) 0.980  Tumor size 1.3(0.9, 2.0) 1.5(1.0, 2.5) 0.155  Number of lymph nodes 5(4, 7) 5(4, 6) 0.693  Bleeding 20(20, 30) 30(20,50) 0.605  Surgical time 83(64, 110) 99(77, 134) 0.149  Postoperative duration 3(3, 4) 6(5, 8) ＜0.001  Drainage time 2(2, 3) 4(3, 6) 0.882  Cost 49043(43659, 55346) 59538(52906, 68769) 0.840  ICU time 0(0, 0) 0(0, 0) 0.969  Time of surgery 0.082  Early start 187 108  Late start 75 28  Gender 0.615  Male 107 52  Female 155 84  Smoking 0.598  Yes 58 27  No 204 109  Alcohol 0.729  Yes 46 22  No 216 114  Comorbidity 0.130  Yes 75 49  No 187 87  Complication 0.650  Yes 15 10  No 247 136  Surgical range 0.606  Lobectomy 143 75  Segmentectomy 75 43  Wedge resection 44 18  Surgical method 0.415  Minimally invasive surgery 259 133  Open surgery 3 3  Surgical approach 0.271  VATS 210 109  UVATS 43 24  RATS 6 0  Open 3 3  Histology 0.797  Benign 23 13  Malignant 239 123  ASA grade 0.667  1 2 2  2 216 109  3 44 25 |

Supplement material 2. Baseline characteristics comparison between low and high total hospital cost group.

| Variables Low cost group (n=199) High cost group (n=199) P |
| --- |
| Age 56(48, 63) 59(50, 66) 0.944  BMI 22.59(21.08, 24.52) 22.86(20.76, 24.57) 0.802  Tumor size 1.2(0.9, 2.0) 1.5(1.0, 2.3) 0.863  Number of lymph nodes 5(4, 7) 5(4, 6) 0.585  Bleeding 20(15, 30) 30(20,50) 0.448  Cost 46353(41937, 49477) 61143(55637, 69405) ＜0.001  Surgical time 76(60, 105) 98(79, 135) 0.160  Postoperative duration 3(3,4) 5(4,6) 0.004  Drainage time 2(2, 3) 3(2, 4) 0.022  ICU time 0(0, 0) 0(0, 0) 1.000  Time of surgery 0.001  Early start 162 133  Late start 37 66  Gender 0.052  Male 89 70  Female 110 129  Smoking 0.038  Yes 51 34  No 148 165  Alcohol 0.062  Yes 41 27  No 158 172  Comorbidities 0.051  Yes 53 71  No 146 128  Complication ＜0.001  Yes 0 25  No 199 174  Surgical range 0.565  Lobectomy 110 108  Segmentectomy 55 63  Wedge resection 34 28  Surgical method 0.685  Minimally invasive surgery 197 195  Open surgery 2 4  Surgical approach 0.225  VATS 155 164  UVATS 37 30  RATS 5 1  Open 2 4  Histology 0.162  Benign 14 22  Malignant 185 177  ASA grade 0.731  1 2 2  2 166 159  3 31 38 |

Supplement material 3. Baseline characteristics comparison between short and long operation time group.

| Variables short surgical time group (n=199) long surgical time group (n=199) P |
| --- |
| Age 56(49, 65) 57(49, 65) 0.110  BMI 22.72(21.01, 24.57) 22.66(20.98, 24.54) 0.162  Tumor size 1.2(0.8, 1.9) 1.5(1.0, 2.5) 0.466  Number of lymph nodes 5(3, 6) 5(4, 6) 0.018  Bleeding 20(20, 30) 20(20,40) 0.183  Surgical time 69(54, 77) 120(100, 150) ＜0.001  Cost 49357(42414, 56746) 55332(49532, 64680) 0.437  Postoperative duration 3(3,5) 4(3,6) 0.752  Drainage time 2(2, 3) 3(2, 4) 0.599  ICU time 0(0, 0) 0(0, 0) 0.414  Time of surgery 0.423  Early start 151 144  Late start 48 55  Gender 0.260  Male 85 74  Female 114 125  Smoking 0.541  Yes 45 45  No 159 154  Alcohol 0.790  Yes 33 35  No 166 164  Comorbidities 0.829  Yes 61 63  No 138 136  Complication 0.535  Yes 11 14  No 188 185  Surgical range 0.121  Lobectomy 118 100  Segmentectomy 50 68  Wedge resection 31 31  Surgical method 1.000  Minimally invasive surgery 196 196  Open surgery 3 3  Surgical approach 0.626  VATS 164 155  UVATS 30 37  RATS 2 4  Open 3 3  Histology 0.727  Benign 19 17  Malignant 180 182  ASA grade 0.446  1 2 2  2 158 167  3 39 30 |
